# Risk Factors for Acute Rheumatic Disease: Exploring Factors at Individual and Collective Levels

**DOI:** 10.1590/0037-8682-0139-2024

**Published:** 2024-11-15

**Authors:** Renata Fonseca Mendoza, Antonio Mutarelli, Bernardo Fonseca Mendoza, José Augusto Almeida Barbosa, Rodrigo Liberato de Oliveira, Bruno Ramos Nascimento, Alexandre Negrão Pantaleão, Isabella Moreira Gonzalez Fonseca, Matheus Assunção Rabello de Oliveira, Airandes de Sousa Pinto, Andrea Beaton, Maria Carmo Pereira Nunes

**Affiliations:** 1 Universidade Federal de Minas Gerais, Faculdade de Medicina, Programa de Pós-Graduação em Ciências da Saúde: Infectologia e Medicina Tropical, Belo Horizonte, MG, Brasil.; 2 Hospital Estadual da Criança, Feira de Santana, BA, Brasil.; 3 Universidade Estadual de Feira de Santana, Feira de Santana, BA, Brasil.; 4 Universidade Federal de Minas Gerais, Faculdade de Medicina, Hospital das Clínicas, Belo Horizonte, MG, Brasil.; 5 Hospital Madre Teresa, Serviço de Hemodinâmica, Belo Horizonte, MG, Brasil.; 6 University of Cincinnati, School of Medicine, Cincinnati Children’s Hospital Medical Center, Department of Pediatrics, Cincinnati, Ohio, USA.

**Keywords:** Acute rheumatic fever, Rheumatic heart disease, Carditis, Risk factors, Outcome

## Abstract

Acute rheumatic fever (ARF) is a complex disease with several clinical manifestations. Its most significant long-term complication is valvular heart damage, commonly referred to as chronic rheumatic heart disease. The risk of ARF varies globally, with over 80% of cases occurring in low- and middle-income countries, highlighting the role of socioeconomic factors. A comprehensive understanding of the risk factors associated with ARF and its clinical, genetic, and sociodemographic mediators can help clinicians identify high-risk individuals, develop effective management strategies, enhance target screening and active case-finding initiatives, and ultimately improve patient outcomes. This review aimed to provide an overview of ARF and its global burden, focusing on the established and potential risk factors associated with its development.

## INTRODUCTION

### ● Overview of acute rheumatic fever

Acute rheumatic fever (ARF) is an immune-mediated inflammatory condition triggered by an abnormal immune response to group A *Streptococcus* (GAS) infections, primarily affecting children and adolescents^1^. This dysregulated immune response can manifest in various major clinical presentations, including inflammation of the heart (carditis), joint inflammation (polyarthritis, polyarthralgia, and monoarthritis), involuntary movements (chorea), distinctive rash (erythema marginatum), and subcutaneous nodules. The most severe and long-lasting complication of ARF is rheumatic heart disease (RHD), which results from recurrent episodes of exacerbated immune activation potentiated by repeated exposure to GAS. This can ultimately lead to heart failure and premature death^2^. Although ARF affects both men and women equally, RHD is more prevalent in women^3^.

Despite advances in medical knowledge and improvements in public health, ARF continues to pose significant challenges for clinicians and researchers^2^ ^,^ ^4^. ARF remains endemic in many low- and middle-income countries as well as in underserved populations in higher-income countries^3^. Understanding the risk factors associated with ARF is crucial for developing targeted prevention and control strategies. This literature review aimed to synthesize the current literature on the contemporary epidemiology of ARF and describe established and potential risk factors, encompassing environmental, genetic, and socioeconomic factors, that contribute to the susceptibility of individuals and populations to ARF. 

PubMed and Excerpta Medica Database (EMBASE) were searched to find recent articles on these topics, followed by backward snowballing. The inclusion and exclusion criteria were determined based on the relevance of the findings and explanations, with a particular emphasis on the methods and results. No time limits were applied, and all selected manuscripts were published in English.

### ● Epidemiology: challenges and global disease burden

***Challenges:*** In contrast to the contemporary data on the prevalence of RHD, a few recent studies have investigated the incidence of ARF (Table 1). Collecting high-quality epidemiological data on ARF poses several challenges. First, diagnosing ARF can be challenging owing to the lack of a specific diagnostic test and the varied clinical presentations of the disease, which can mimic several pathological conditions. Current diagnostic criteria, such as the Jones criteria, may not be universally applicable or sufficiently sensitive, leading to underdiagnosis and underreporting. Second, implementing the Jones criteria globally is difficult due to resource constraints, preventing the assessment of GAS antibody titers and the completion of necessary laboratory tests or cardiac evaluations (ECG and echocardiography), and the lack of expertise in an accurate clinical evaluation. Third, evidence indicates low awareness among healthcare workers and communities regarding ARF and its association with the GAS and RHD^5^. 

**TABLE 1:** Global prevalence of acute rheumatic fever reported in recent studies.

Study	Region	Age range	Prevalence (per 100,000)	Comment
Beaudoin, 2015^32^	America Samoa	<18	2.5	Peak incidence at the age of 11 years: 3.2 per 100,000
Katzenellenbogen, 2020^33^	Australia	5-14	11.2	Indigenous population prevalence: 136.4 per 100,000
Okello, 2021^34^	Uganda	5-14	25	Prevalence of any ARF attack: 47.9 per 100,000
Gürses, 2021^35^	Turkey	5-15	8.84	Eastern Anatolia Region prevalence: 14.4 per 100,000
Lindholm, 2023^36^	Australia	5-14	0.77	Pacific Islander prevalence: 32.1 per 100,000

The epidemiology of ARF varies widely across countries, reflecting the complex interplay between genetic, socioeconomic, and environmental factors. Although the incidence of ARF has decreased in high-income countries over the past century, it remains a major public health concern in many low- and middle-income countries, particularly in sub-Saharan Africa, South Asia, and the Pacific region^4^. This trend is consistent with the disease burden and mortality observed in RHD^2^. Additionally, available data suggest that the incidence of ARF remains disproportionately high among indigenous populations and socioeconomically disadvantaged communities, even in high-income countries^6^.

### ● Age and sex

The first episode of ARF typically occurs in children aged 5-15 years^1^. Recurrent episodes more commonly affect older children and adolescents, with limited precise data on the upper age limit; however, such episodes rarely occur after the age of 25-30 years. The prevalence ratio of ARF between men and women remains unclear, although most evidence suggests an equal prevalence in both genders^7^. However, local estimates and hospital registries have suggested a slightly higher prevalence in women.

***Global prevalence:*** The global mean incidence of ARF is 19 per 100,000 school-aged children, with estimates ranging from 8 to 51 per 100,000 people^1^ ^,^ ^2^. The incidence is lowest in developed countries (e.g., ≤2 cases per 100,000 school-aged children in the United States). However, limited data exists regarding the vulnerable sub-populations^8^. Notably, Indigenous Australian children are particularly affected, with incidence rates ranging from 153 to 380 cases per 100,000 children, possibly due to the more frequent diagnoses compared with other regions. In the rural and semi-urban areas of Africa, the incidence rate can be as high as 25 cases per 100,000 people^9^. However, large-scale and reliable registries are still being developed.

Over the last five decades, the incidence of ARF has significantly declined in high-income regions from an epidemiological standpoint. However, this progress has not been universal as poorer endemic regions have experienced stable or increasing trends. A similar trend was observed for RHD from 1990 to 2019, with age-standardized prevalence often increasing in low- and middle-income areas despite a global reduction in mortality and disability-adjusted life years^2^. Furthermore, in recent decades, the overall difference between low- and high-incidence settings has increased both in terms of incidence and prevalence, serving as indicators of the impact of sociodemographic variables^2^.

### ● Risk factors for ARF

**FIGURE 1:**
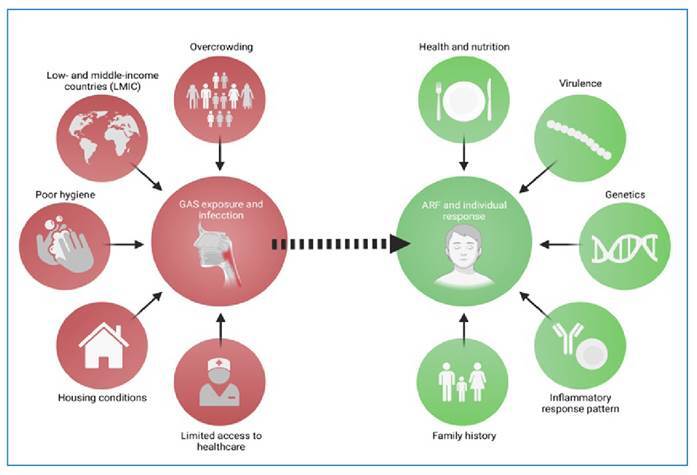
Overview of the risk factors associated with acute rheumatic fever. The risk factors are categorized into those related to group A *Streptococcus* (GAS) exposure and infection, as well as those associated with individual susceptibility to develop the disease. The inflammatory response to GAS infection plays an important role in the development of the disease.

ARF is a multifaceted condition triggered by GAS infection, involving various factors that contribute to the causal pathways leading to GAS exposure in RHD (Figure 1). The risk of ARF at both individual and population levels is determined by a complex interplay of environmental, host, and bacterial factors^10^. These factors collectively influence susceptibility to the disease and its subsequent outcomes^11^.

The ARF risk depends on three primary determinants: (i) environmental factors, (ii) socioeconomic factors, and (iii) individual susceptibility. Understanding the relative contributions of environmental risk factors can be challenging due to their overlapping nature and association with socioeconomic disadvantages, particularly in low-income settings. Conversely, at the individual level, predisposing factors encompass the patient’s susceptibility, bacterial characteristics, and the dynamic interplay between the host and bacteria.

### ● Environmental risk factors

***Household factors and living conditions:*** Household crowding has consistently played a central position in studies investigating the risk factors for ARF and RHD and has emerged as a significant contributing factor. Previous studies have extensively explored various environmental, socioeconomic, and housing-related living conditions^11^. The significance of household crowding as a risk factor for ARF stems from its role in increasing the effective reproduction number of infections within households, which has also been linked to an increased risk of other bacterial diseases. Nevertheless, although some studies did not find a significant association between the number of individuals per bedroom and household size and GAS-positive sore throats, a well-designed study employing multivariate analyses revealed a notable positive correlation between crowding and pharyngeal GAS carriage^12^.

Inadequate washing facilities and limited resources can contribute to an increase in bacterial load on the skin of household members, consequently elevating the transmission rates and the likelihood of developing related skin and pharyngeal infections^11^. Indeed, hygiene is recognized as a crucial determinant of the development of GAS pyoderma, which can potentially cause ARF^11^. However, studies examining risk factors have not assessed the influence of household washing and laundry facilities on ARF or RHD.

Cold, damp, and moldy homes are associated with adverse respiratory health outcomes. Such environmental conditions may also create conditions that facilitate the transmission of bacterial pathogens such as GAS^13^. Moreover, household resources, including the type of ceiling and wall materials, have been investigated as risk factors for subclinical RHD, although they often overlap with low-income housing conditions^14^.

***Tobacco:*** Environmental tobacco smoke has been identified as a contributing factor to elevated GAS transmission and susceptibility to infection, thereby amplifying the risk of ARF^15^. In a previous study, 71% of patients with ARF reported cohabiting with smokers^15^.

### ● Socioeconomics risk factors

The risk of ARF closely correlates with socioeconomic and health conditions, especially in terms of access to basic healthcare provided at the primary level, such as health promotion, primordial prevention, and prophylaxis. In disadvantaged environments, the prevalence of ARF is significantly higher than that in better-resourced settings. Evidence from various regions including New Zealand (with risks up to 26 times higher for children in the most deprived areas), Oceania, Uganda, Brazil, and India supports this correlation (Table 2). The association between low socioeconomic status and ARF is likely environmental, driven by the chronic risk of frequent superficial GAS exposure and related to access to basic healthcare services. 

**TABLE 2:** Socioeconomic risk factors of acute rheumatic fever.

Study	Design	Country/region	Sample size	Socioeconomic relation with ARF
Chun, 1984^37^	Retrospective observational	USA	104	Low socioeconomic status
Vlajinac, 1991^38^	Case-control	Yugoslavia	148	Home dampness and low maternal education
Gurney, 2016^39^	Retrospective observational	New Zeland	711	Deprivation strata
Elthohami, 2016^40^	Prospective	Arabia Gulf Country	86	Limited healthcare access
Cannon, 2019^41^	Cohort	UK	17,416	Overcrowded household
Loizaga, 2021^42^	Chart Review	USA	947	Living in deprived communities

Limited access to healthcare services is strongly correlated with a low socioeconomic status, which, in turn, is associated with a higher prevalence of ARF. Furthermore, parents’ educational levels significantly contribute to the development of ARF, primarily due to challenges related to raising awareness about various aspects of the disease. These challenges include inadequate prevention efforts, delayed or missed diagnoses, and the lack of understanding regarding the importance of adequate treatment.

***Healthcare:*** Understanding ARF and its prevention through proper healthcare utilization by treating sore throat infections is widely recognized as essential for primary prevention^16^. However, the timing of treatment for GAS infections remains controversial. Two randomized clinical trials comparing immediate versus delayed (48 to 56 hours) antibiotic treatment for GAS pharyngitis suggested that immediate antibiotic treatment may suppress the immune response, potentially increasing susceptibility to relapse and recurrence of GAS pharyngitis^17^.

A recent systematic review and meta-analysis highlighted that although antibiotic treatment for pharyngeal GAS infections often leads to high culture conversion rates within 24 hours, a significant proportion of patients (9.1%, 95% confidence interval: 7.3-11.3) still tested positive for GAS culture after completing therapy^18^. This finding underscores the need for further exploration of the optimal timing of the initiation, dosing, and maintenance of therapy. Nevertheless, given that ARF primarily occurs in high-risk populations, health education on the timely treatment initiation remains of paramount importance for healthcare providers.

Despite these challenges, a structured review of primary interventions aimed at reducing the incidence of GAS infections and ARF revealed limited evidence supporting the effectiveness of primary prevention strategies in lowering ARF rates^16^. The two key primary intervention strategies recommended for high-risk communities include establishing school-based clinics to identify and treat GAS pharyngitis and skin infections. Administering injectable benzathine penicillin G is recommended for children with either positive results on GAS throat culture or exhibit a high clinical suspicion of GAS infection when a throat culture is not available^16^.

***Income:*** Individuals from socioeconomically disadvantaged families are more susceptible to ARF due to factors such as poor living conditions, inadequate hygiene practices, limited healthcare access, and insufficient treatment of streptococcal infections^11^ ^,^ ^15^. Overcrowding, lack of access to clean water, and inadequate sanitation further exacerbate the risk of streptococcal infection. Consequently, some risk factors associated with low-income environments overlapped with those associated with impoverished settings.

Low-income individuals may also encounter challenges related to poor nutrition and other health conditions that can compromise their immune system, potentially increasing the risk of infection^19^ ^,^ ^20^. Although ethnicity is sometimes considered a contributing factor to ARF, the increased vulnerability reported in specific ethnic groups may be more precisely linked to the higher rates of poverty and overcrowding rather than genetic predisposition. Nevertheless, uncertainties persist, as familial studies have shown significantly higher rates of echo-detected RHD among the relatives of patients with advanced-stage disease, compared with unrelated individuals living in the same household^21^. This suggests that ARF follows a similar pattern, as indicated by studies comparing the prevalence of ARF in individuals with and without a family history of the disease.

### ● Individual risk factors

***Host factors:*** The risk of ARF is significantly influenced by specific demographic factors such as age and ethnicity. Nonwhite ethnicity was associated with a higher risk of developing ARF. Nutritional status is another important risk factor associated with socioeconomic deprivation^22^. Malnutrition in early life leads to an imbalance in the immune system, which is believed to increase the risk of developing autoimmune diseases^22^.

Poor oral health, especially early childhood caries, is significantly associated with the low socioeconomic status of parents, which may potentially contribute to the risk of ARF through various mechanisms^11^. Furthermore, a previous case-control study identified a significant association between high intake of sugar-sweetened beverages and increased risk of ARF in a multivariate analysis^23^.

Inherited genetic variations significantly contribute to ARF susceptibility^24^. Although the precise genetic mechanisms responsible for ARF susceptibility and disease progression remain unclear, significant advancements have been made in identifying the potential genetic factors that contribute to the development of ARF. Numerous susceptibility loci have been documented, particularly for genes associated with the immune system. This suggests that the underlying mechanisms are influenced by multiple genes, reflecting a diverse and complex genetic interplay.

In addition, a comprehensive analysis of more than 400 pairs of twins from six studies provided further evidence supporting the genetic predisposition and heritability of ARF^25^. The analysis showed a 44% risk of ARF in monozygotic twins and a 12% risk in dizygotic twins^25^, with an odds ratio of 6.39 highlighting the strong association between zygosity and concordance. Furthermore, the analysis indicated a heritability of 60%^25^. Case-control studies have further substantiated this finding, showing that individuals with a positive family history of ARF have a five-fold higher risk of ARF. This observation aligns with the similarities noted in first-degree relatives of patients with established RHD who undergo echocardiographic screening^21^ ^,^ ^23^ ^,^ ^24^.

Several genetic polymorphisms are significantly associated with ARF. The human leukocyte antigen (HLA) system, which plays a role in immune recognition and response, has been extensively investigated. Various associations between HLA class II antigens and ARF susceptibility have been identified across different populations, with the most robust associations observed for HLA-DR and HLA-DQ alleles^26^ ^,^ ^27^. However, these associations may vary across ethnic groups, geographical regions, and disease outcomes. Notably, a stronger and more robust association was observed with RHD, highlighting the complex and heterogeneous nature of ARF. Interestingly, the associations with HLA class II alleles appeared to be more consistent in patients with relatively homogeneous clinical manifestations, similar demographic profiles, and late valvular involvement^27^. In addition to HLA associations, several non-HLA genes have been implicated in ARF susceptibility and severity, affecting various aspects of the immune response, including cytokine production, immune cell activation, and pathogen recognition^27^.

The inflammatory response initiates the pathogenic process of ARF; however, it may not always drive its progression over time. This assumption is supported by the presence of autoantibodies in healthy individuals and the fact that cardiac myosin, a target of autoimmunity in ARF, is rarely expressed on cell surfaces. Therefore, understanding the function of host variables, particularly genetic susceptibility, is crucial for fully comprehending the genesis and progression of ARF^28^.

***Bacterial factors:*** Bacterial factors significantly contribute to the development of ARF and influence the frequency of GAS infections in individuals with and without ARF^29^. Certain GAS strains possess virulence factors that increase the likelihood of ARF by triggering a strong immune response in the host^29^. Additionally, a history of recurrent GAS infections, such as pharyngitis or impetigo, increases the risk of developing ARF owing to repeated exposure to the pathogen^5^. 

***Host-bacterial interaction:*** The primary mechanism underlying autoimmunity in ARF involves molecular mimicry^1^ ^,^ ^30^ ^,^ ^31^. This is due to the significant similarity between the M protein and carbohydrate antigen epitopes observed in both the GAS bacterium and human cardiac myosin and laminin. Cross-reactive antibodies bind to the valve surface and enhance the expression of molecules that facilitate the attachment and infiltration of specific immune cells^6^ ^-^ ^8^. This process leads to the release of inflammatory substances and the reduction in the levels of certain anti-inflammatory compounds. Consequently, the proliferation of these molecules leads to the recognition of other self-proteins, exacerbating the immune-mediated damage^31^. Inflammation triggers the formation of new blood vessels and scarring, which evolves into fibrosis and calcification, contributing to the typical pattern of valvular involvement observed in RHD^9^. Additionally, antibodies targeting specific bacterial components may interact with brain cells, causing an excessive release of dopamine and resulting in chorea. The accumulation of immune complexes also contributes to the temporary and migratory joint symptoms associated with ARF.

Besides molecular mimicry, several other hypotheses explain the development of ARF. One hypothesis suggests that the streptococcal M protein binds to type IV collagen, potentially triggering an immunological reaction that damages the heart valves. Although molecular mimicry is important for understanding ARF development, evidence suggest that antibodies against collagen may play a role in disease progression^31^. 

## FINAL CONSIDERATION

ARF continue to pose significant health challenges in numerous regions worldwide, particularly in low-resource settings. Various risk factors associated with the development of ARF have been identified. However, the precise mechanisms and the interplay between these factors remain unclear. Furthermore, a comprehensive understanding of the factors linked to ARF is essential for improving clinical outcomes. This knowledge can guide the development and refinement of health policies regarding education and health awareness; early detection and prevention; and adherence to prophylaxis, treatment, and follow-up. Collaborative research on RHD and ARF has the potential to improve outcomes and support disease eradication.
